# The effects of thermal capsulorrhaphy of medial parapatellar capsule on patellar lateral displacement

**DOI:** 10.1186/1749-799X-3-45

**Published:** 2008-09-30

**Authors:** Naiquan Zheng, Brent R Davis, James R Andrews

**Affiliations:** 1 University of North Carolina at Charlotte, Charlotte, NC, USA; 2 American Sports Medicine Institute, Birmingham, AL, USA

## Abstract

**Background:**

The effectiveness of thermal shrinkage on the medial parapatellar capsule for treating recurrent patellar dislocation is controversial. One of reasons why it is still controversial is that the effectiveness is still qualitatively measured. The purpose of this study was to quantitatively determine the immediate effectiveness of the medial parapatellar capsule shrinkage as in clinical setting.

**Methods:**

Nine cadaveric knees were used to collect lateral displacement data before and after medial shrinkage or open surgery. The force and displacement were recorded while a physician pressed the patella from the medial side to mimic the physical exam used in clinic. Ten healthy subjects were used to test the feasibility of the technique on patients and establish normal range of lateral displacement of the patella under a medial force. The force applied, the resulting displacement and the ratio of force over displacement were compared among four data groups (normal knees, cadaveric knees before medial shrinkage, after shrinkage and after open surgery).

**Results:**

Displacements of the cadaveric knees both before and after thermal modification were similar to normal subjects, and the applied forces were significantly higher. No significant differences were found between before and after thermal modification groups. After open surgery, displacements were reduced significantly while applied forces were significantly higher.

**Conclusion:**

No immediate difference was found after thermal shrinkage of the medial parapatellar capsule. Open surgery immediately improved of the lateral stiffness of the knee capsule.

## Background

Recurrent patellar dislocation can be the result of abnormal anatomy, such as trochlear dysplasia, patellar alta, soft tissue imbalance, or malalignment of the quadriceps extensor mechanism [1,2]. Strong joint capsule and tissue surrounding the patellar keep the patella at the center of the trochlear groove. If the joint capsule and surrounding tissue of the patella is not balanced, this will cause the patella to be translated to one side or onto the edges of the trochlear groove as the knee flexes and extends. A recent cadaveric study showed that the patellar translated medially 4 mm to engage the trochlear groove at 20° knee flexion, then translated to 7 mm lateral by 90° knee flexion [3]. It is important for the patella to engage the trochlear groove before further knee flexion and to prevent dislocations.

Tissue shrinkage has been used to alter mechanical properties of soft tissues in order to regain lost function. Shoulder capsular shrinkage was proposed a few years ago as a therapeutic modality in a select group of patients with instability in 1999 [4]. A number of early clinical studies described promising outcomes [5,6]. Reports of outcomes from later, prospective studies of shoulder with a wide spectrum of diagnoses have been more mixed [7-9]. Although there are several reports in the literature of thermal capsulorrhaphy used to treat instability in the ACL [10-13], only one paper was found reporting clinical use of the thermal capsulorrhaphy to treat recurrent patellar instability and subluxations [14]. The basic science of laser- and radiofrequency-induced capsular shrinkage has been studied extensively [15-22]. The objective of this study was to focus on human joint capsule and develop a quantitative measure of its effectiveness for clinical application. We hypothesized that after medial shrinkage of the medial parapatellar capsule the lateral translation of the patella would be significantly reduced. The lateral translation and stiffness of the knee capsule in a simulated physical exam were compared among healthy subjects, cadaver knees before and after medial shrinkage, and cadaver knees post open surgery (open medial reefing of the medial parapatellar capsule and retinaculum). Our purpose was to test our hypothesis and set up a testing protocol for future clinical studies.

## Methods

Nine fresh-frozen cadaver specimens were used for the study. The average age at death of the three males and six females was 65 years (range, 62 to 77 years). The specimens were 5 right and 4 left knees without any visible deformity or abnormality. They were sectioned about 20 cm proximal and distal from the joint line. Both tibia and femur were secured in polyvinyl chloride (PVC) pipes during tests. The specimen was mounted in a custom-made frame. The adjustable frame allowed the specimen to be mounted in any position and no preload was applied to the joint. To simulate the tension of the quadriceps tendon, a tension of 18 N was applied to the tendon using a spring scale.

In order to test our testing protocol, ten healthy subjects were recruited and tested on both legs. The 4 females and 6 males averaged 27 years of age. Data from healthy subject may provide the norm data of lateral stiffness of the knee capsule for future patients. The protocol had been approved by the local Institutional Review Board. In a pilot study, knees of both specimens and subjects were tested at different flexion angles. Although patellar dislocation is often occurred at 20° knee flexion, our pilot study showed similar lateral displacement and stiffness on cadaveric knees when tested at 0° and 20°. However, the healthy subjects' data was more repeatable and reliable when tested at 0°. This will be true for data collection from patients before and after medial shrinkage in future. Based on the results, full extension of the knee was chosen as the best angle for testing reliability and repeatability.

**Figure 1:**
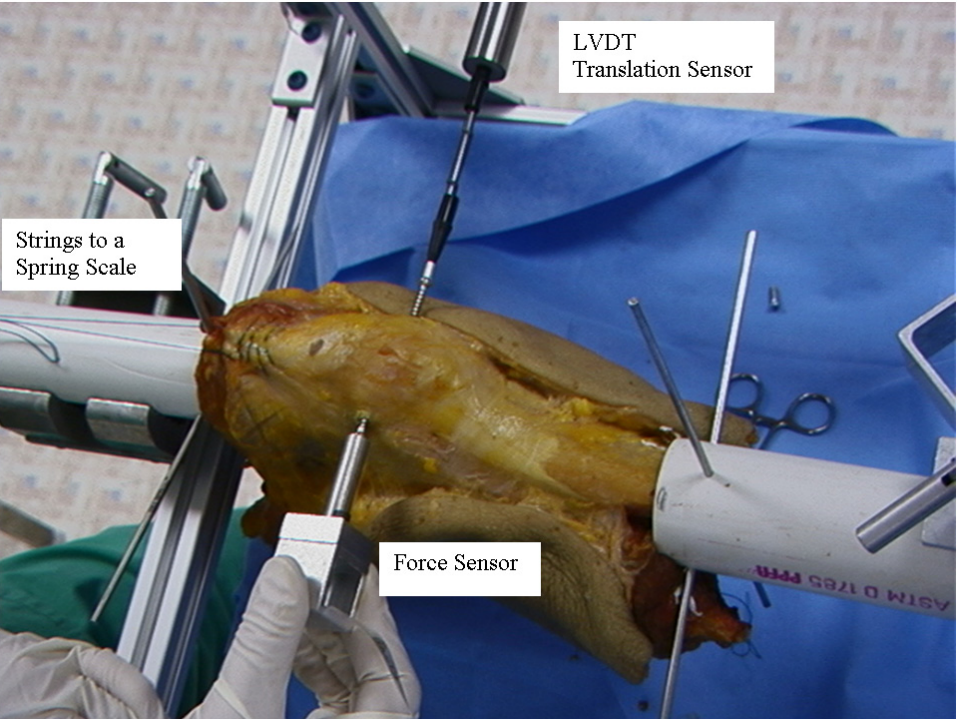
**Test set-up.** A LVDT translation sensor was mounted on the lateral side and a force sensor was mounted on an adaptor to apply compressive force from the medial side of the patella.

**Figure 2:**
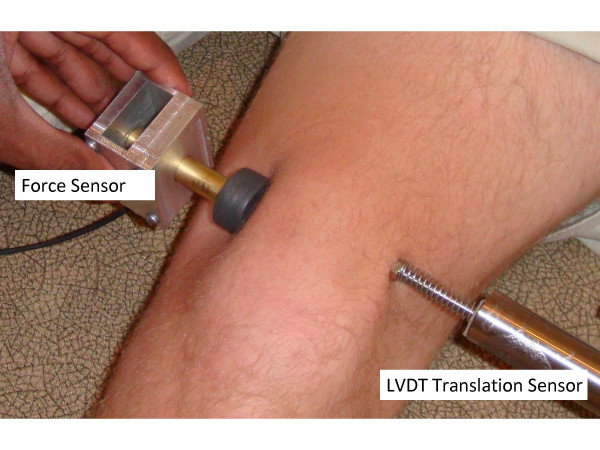
**Test set-up for healthy subjects.** A LVDT translation sensor was mounted on the lateral side and a force sensor was mounted on an adaptor to apply compressive force from the medial side of the patella.

The testing set-up was designed to be able to use on both healthy subject and cadaver knees. Lateral translation of the patellar was recorded using a linear variable displacement transducer (LVDT) translation sensor (Macro Sensors, Pennsauken, NJ) (Figure 1). The lateral force applied to the patellar was recorded using a Flexiforce force sensor (Tekscan, South Boston, MA). A custom-made adaptor was used to hold the sensor and apply force. Both the force and displacement sensor were calibrated before and after use. The error of the force sensor was less than 0.5 N with good repeatability (variation <2.5%). The error of the LVDT sensor was less than 0.1 mm with high repeatability (variation <0.01%). These sensors had high repeatability. Forces were applied from medial side of the patella. A screw was mounted on the medial side of the patellar for cadaveric knees. Both force and translation sensors were connected to a computer via a data acquisition board (Data Translation, Boston, MA). Both force and displacement data were displayed real-time on the computer screen. Data were collected and stored for further analysis. Each test was repeated three times. For healthy subjects they were instructed to lie on an exam table and to relax during the test. Forces were applied to the medial side of the patella (Figure 2). Before data collection a baseline of applied force was first set by the examiner when the patella started to tilt or rotate. The force and displacement were monitored by the examiner at real-time through the computer display. A rubber disk was mounted to the force adaptor. No screw was mounted on the patella of healthy subjects since the physician was able to easily apply force without slippage of the force adaptor on the medial border of the patella.

**Figure 3:**
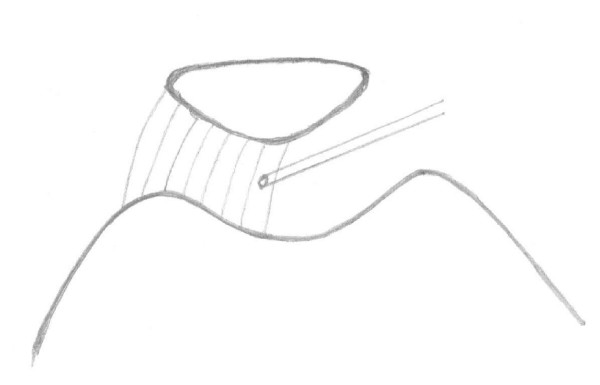
Thermal energy was applied in a paint-brush fashion medially from patella on the inner surface of the medial parapatellar capsule.

After initial testing, the knee capsule thermal modification was performed on cadaver specimens using Mitek VAPR (DePuy Mitek, Norwood, MA). Arthroscopy and thermal shrinkage of the cadaver knee was applied to the medial portion of the capsule by a surgeon according to clinical protocol and manufacture setting (65°C and 40 Watts). Mitek end effect temperature control electrodes were used. Thermal energy was applied in a paint-brush fashion medially from patella on the inner surface of the medial parapatellar capsule (Figure 3). The biomechanical testing was repeated after the process. In the end, each cadaver knee was used for the mini-open medial reefing after thermal capsulorrhaphy. A 4 cm incision, starting at the superior pole of the patella, was created 2 cm medial extending distally. The incision was parallel to the medial border of the patella. Mini-open medial reefing of the medial capsular structure and retinaculum was conducted following the procedure previously described [23]. The medial retinaculum and other structures were shortened about 5 mm in medial lateral direction. The testing was repeated once again after open surgery.

Under each condition, testing was repeated three times. Measures were averaged from three trials and analyzed. Repeated measure analysis of variance (ANOVA) was used to compare the differences between thermal shrinkage and medial reefing and to compare differences between healthy subjects and cadaver knees before treatments. The p-value was set at 0.05 with a power level of 0.8 for statistical significance.

## Results

### Healthy Subjects

**Figure 4:**
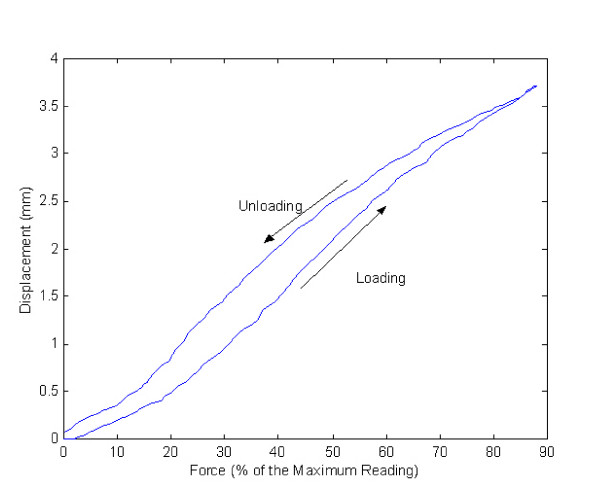
**A typical loading and unloading curve.** Y axis is the displacement in mm and X axis is the force applied in % of the maximum reading calibrated.

The average lateral translation ± standard deviation was 10.5 ± 4.0 mm for the males and 10.9 ± 3.8 mm for the females. No significant differences were found between genders or between left and right knees. The average force applied ± standard deviation was 19.5 ± 4.8 N for the males and 15.2 ± 3.9 N for the females. Male subjects showed higher stiffness than female, with higher force required for the same displacement (Table 1). Stiffness was slightly higher in the right knees for males and in the left knees for females, but neither difference was significant (Table 2). Figure 4 shows a typical loading and unloading curve during test. The stiffness was determined by dividing the peak force applied by the corresponding displacement at the peak force.

**Table 1:** Force, displacement and stiffness for healthy subjects (mean ± standard deviation)

Gender	Force (N)	Displacement (mm)	Stiffness (N/mm)
Male	19.5 ± 4.8	10.5 ± 4.0	2.16 ± 0.98
Female	15.2 ± 4.0	10.9 ± 3.8	1.59 ± 0.77

**Table 2:** Force, displacement and stiffness of left and right knees by gender (mean ± standard deviation)

Gender	Knee	Force (N)	Displacement (mm)	Stiffness (N/mm)
Male	Left	17.4 ± 4.6	10.9 ± 4.2	2.04 ± 0.93
	Right	21.6 ± 4.7	10.5 ± 4.3	2.29 ± 1.16

Female	Left	15.8 ± 4.6	10.9 ± 4.4	1.74 ± 0.98
	Right	14.4 ± 3.8	10.8 ± 2.5	1.44 ± 0.89

### Cadaver knees

The average lateral translation ± standard deviation for cadaver knees before thermal shrinkage was 10.5 ± 1.9 mm, after thermal shrinkage 10.9 ± 1.9 mm, and 5.7 ± 1.9 mm after medial reefing. No significant differences in peak force, peak displacement or stiffness were found between knees of healthy subjects and cadavers (Table 3). Significant differences were not found between before and after thermal shrinkage in cadaver knees. Cadaver knees after open surgery showed significantly higher stiffness than before thermal shrinkage and after thermal shrinkages (p < 0.001). Stiffness was not significantly different among healthy subjects and cadaver knees before and after thermal shrinkage. The forces applied to the healthy subjects were significantly lower than that applied to the other three groups: cadaver knees after open surgery (p < 0.001); cadaver knees before thermal shrinkage (p = 0.004); and cadaver knees after thermal shrinkage (p = 0.007).

**Table 3:** Force, displacement and stiffness (mean ± standard deviation)

Group	Force (N)	Displacement (mm)	Stiffness (N/mm)
Healthy subjects	17.6 ± 4.8	10.7 ± 3.7	1.91 ± 0.92
Cadaver before thermal shrinkage	23.2 ± 4.5	10.5 ± 1.9	2.27 ± 0.54
Cadaver after thermal shrinkage	22.9 ± 3.2	10.9 ± 1.9	2.15 ± 0.43
Cadaver after open surgery	25.3 ± 3.9	5.7 ± 1.9	5.10 ± 2.53

Force applied was not significantly different among the cadaver treatment groups. Cadaver knees after open surgery showed significantly less displacement than the other three groups: less than healthy subjects (p < 0.001), less than cadaver knees before thermal shrinkage (p < 0.001), and less than cadaver knees after thermal shrinkage (p = 0.002). No significant differences in displacement were found among healthy subjects, cadaver knees before and after thermal shrinkage.

## Discussion

The purpose of this study was to focus on the possible biomechanical testing of patellar instable patient before and after thermal shrinkage of medial parapatellar capsule. Our test set-up allowed us to record force applied and lateral displacement of patellar during a simulated physical exam on both healthy subjects and cadaveric knees. The force applied, lateral displacement of the patella and the stiffness of the medial parapatellar capsule were compared among the healthy subjects, cadaveric knees before thermal shrinkage, cadaveric knees after thermal shrinkage and open surgery. The test set-up was capable to quantify the force applied, lateral displacement of the patella during physical exam of healthy subject and can be used in future studies of evaluating the effectiveness of thermal shrinkage of medial parapatellar capsule in patients with recurrent dislocation of patella. The study did not find significant changes of the medial parapatellar capsule in resisting lateral force.

Patellar kinematics of cadaveric knees has been studied extensively. Three-dimensional patellar movement during knee flexion and extension has been studied in vitro using cadaveric knees [3]. The medial and lateral translation of the patella was about 4 mm medial from full extension to 20° flexion, about 7 mm lateral from 20° to 90° flexion. The initial 4 mm medial translation is very important to prevent patella from dislocation laterally. A tight medial parapatellar capsule may contribute the medial translation at initial knee flexion. Thermal shrinkage of medial parapatellar capsule may improve its stiffness and capacity in resisting lateral dislocation of the patella. Although the basic science of laser- and radiofrequency-induced capsular shrinkage has been studied extensively [15-22], in recent prospective studies of shoulder with a wide spectrum of diagnoses, the effectiveness of thermal capsulorrhaphy has been mixed [7-9]. It is important to quantify the effectiveness of thermal capsulorrhaphy in clinic. This study investigated the feasibility of biomechanical testing of the medial parapatellar capsule in living subjects and cadaveric knees. The test set-up could be used on patients with recurrent dislocation of patellar before and after thermal capsulorrhaphy in future.

The medial patellofemoral ligament (MPFL) plays a major role in patellar stability [24,25]. The MPFL consists of a thickened band of tissue originating from the medial epicondyle and inserting on the superior half of the patella. Nomura et al measured the increased laxity resulting from cutting the MPFL [26]. They applied a 10 N tension on the quadriceps and a 10 N lateral displacing force. The lateral displacement of the patella increased from 6 mm for the intact knee to 13 mm after cutting the MPFL. Hautamaa et al applied 9 N to the quadriceps and a lateral displacing force of 22 N to the patella [27]. They found a mean patellar displacement of 9 mm, which is similar to our results. We applied a tensile force of 18 N to the quadriceps to simulate the tension at rest. Our applied force to cadaveric knees averaged about 23 N which is similar to their lateral displacing force of 22 N. The thermal effect on the MPFL is unknown. In this study, we did not monitor the temperature change along the depth of the tissue.

Six degrees of freedom patellar tracking during first 15° voluntary knee flexion has been studied in vivo using optoelectronic motion capture system with a small patellar clamp [28]. In a pilot study we followed their procedure on 3 healthy subjects and found the lateral translation measure in six degrees of freedom was almost identical from LVDT sensor, the other two translations were less than 2 mm, and three rotations was less than 3 degrees. But the procedure was very complicated and the patellar clamp was difficult to stay still relative to the patella. In a full extension position a subject lying on an exam table was much easier to be relaxed than a flexed knee position, which produces minimal influence to the lateral displacement by the quadriceps. Cadaveric knee data also demonstrated similar lateral displacement and stiffness between full extension and 20° knee flexion. Both male and female subjects demonstrated similar lateral displacements during physical exam, though higher forces were applied to the males. Our results also show that there were no significant mechanical differences between live subjects and fresh cadavers. This data may be useful in estimating the probable effects of thermal shrinkage on the knee capsule in patients.

Effectiveness of thermal shrinkage has been studied at length in animal models [15,16,22,29]. Studies using animal specimens found ultrastructural alterations including a general increase in cross-sectional fibril diameter and loss of fibril size variation. Thermally induced ultrastructural collagen fibril alteration is likely the predominant mechanism of tissue shrinkage caused by application of radiofrequency energy. Over the last two to three years, arthroscopic thermal capsulorrhaphy for treatment of shoulder instability has undergone vigorous examination [7,30-38]. Although the short-term outcomes of shoulder capsule shrinkage did not show significant difference than those without capsular shrinkage, long-term outcomes of thermal shrinkage for baseball pitchers are much better. Dugas and Andrews [39] reported an approximate 20% improvement in the rate of return to play with the addition of thermal capsular shrinkage to traditional treatments. Reinold et al. [40] studied the return-to-competition rate and functional outcome of overhead athletes following arthroscopic thermal-assisted capsular shrinkage. They followed 130 overhead athletes and found 87% successfully returned to competition with good-to-excellent long-term results. However, recently there are reports of glenohumeral chondrolysis after shoulder arthroscopy with thermal capsulorrhaphy [32,41,42]. Excessive heat from the procedure may have led to chondral damage and further research is needed to prevent this complication.

Coons and Barber treated 53 knees with a combination of capsule shrinkage and lateral release and followed them for an average of 53 months [14]. Outcome was measured using the Lyscholm and Fulkerson knee scores, physical exam and the visual analog score. Subjectively, 90% of the patients reported excellent or good results. These results suggested that thermal capsule shrinkage may be valuable in treating the instable patella. However no detail of the thermal capsule shrinkage was reported regarding the temperature and power used. According to animal studies the amount of shrinkage potential was directly related to the temperature of the probe, the time of application, and the tissue quality [4,20,21,43]. We used the intact cadaver knee joint and applied 65°C 40 watts as suggested by the manufacture. We did not find post-treatment changes of lateral displacement and stiffness. This could be due to the old age of the specimens. As a result of decreasing quantities of heat-sensitive bonds between type 1 collagen molecules, the potential for shrinkage decreases with increasing age. The decreased tissue stiffness of isolated tissue by thermal shrinkage may be accountable for unchanged displacement [20]. The treated tissue began to show signs of healing by 6 weeks and the tissue stiffness returned to normal by 12 weeks [20].

Mini-open medial reefing and arthroscopic lateral release have been used to treat recurrent patellar dislocation [23,44-46]. Good clinical outcomes have been reported with improved knee mobility and daily function. After mini-open medial reefing, the lateral displacement was reduced to 53% and the stiffness against the lateral force was over two times when compared with pre-reefing data. Our results confirmed the immediate effectiveness of medial reefing and matched these reported clinical data.

The limitations of this study are that the specimens were fresh-frozen and thawed over 24 hours, and they came from people over 60 years of age and the patella may not be instable. The influences of freezing on tissue response to thermal energy may be more significant than we expected. The temperature and power applied were set by the manufacture for clinical application. It was not the purpose of this study to investigate the influences of applied temperature and power, which have been done extensively in animal studies. Although we evaluated several knee flexion angles in our pilot study, for the full study we only tested at full extension of the knee to reduce the number of factors affecting our data collection in future clinical studies.

This study measured the immediate effect of applying thermal energy to the medial parapatellar capsule of human cadaver knees. We found that the fresh-frozen cadaver knees were similar in biomechanical properties of lateral displacement and stiffness to healthy young adults. The application of thermal energy to the medial capsular structures of human cadaver knees produced no statistically or clinically appreciable differences in medial structure stiffness compared to pre-treatment values. This study suggests that there is no need to test the patellar stability right after treatment for future clinical studies. The testing protocol worked fine with human subjects and cadaver knees. After proper post-operative immobilization and tissue healing, it is possible that this procedure may provide a reasonable alternative to open surgery for the treatment of patellar instability. Further clinical study is needed to investigate the long-term effect of thermal modification on the knee capsule.

## Conclusion

No immediate difference in lateral displacement and stiffness was found after medial shrinkage. Open surgery immediately improved the lateral stiffness of the knee capsule. This study developed a non invasive technique to quantify the effectiveness of medial shrinkage on human knees. The long-term effect of the treatment need to be further studied.

## Competing interests

The authors declare that they have no competing interests.

## Authors' contributions

NZ carried out the study design, test set-up, data acquisition, analysis and interpretation of data, performed statistical analysis and draft of the manuscript. BD performed surgeries, data acquisition, participated in the design of the study and helped to draft the manuscript. JA made substantial contribution to conception and design of the study, provided guidance of the surgical procedures, and helped to draft the manuscript. All authors read and approved the final manuscript.
